# The socioeconomic impact of tuberculosis on children and adolescents: a scoping review and conceptual framework

**DOI:** 10.1186/s12889-022-14579-7

**Published:** 2022-11-23

**Authors:** S. Atkins, L Heimo, DJ Carter, M. Ribas Closa, L. Vanleeuw, L. Chenciner, P. Wambi, K. Sidney-Annerstedt, U Egere, S Verkuijl, A Brands, T Masini, K Viney, T. Wingfield, K Lönnroth, D. Boccia

**Affiliations:** 1 WHO Collaborating Centre On Tuberculosis and Social Medicine, Department of Global Public Health, Karolinska Institutet, Stockholm, Sweden; 2 Faculty of Social Sciences, Tampere University, Tampere, Finland; 3 Faculty of Public Health and Policy, London School of Hygiene and Tropical Medicine, London, UK; 4 Health Systems Research Unit, South African Medical Research Council, Cape Town, South Africa; 5 Royal Free London NHS Foundation Trust, London, UK; 6 Uganda Tuberculosis Implementation Research Consortium, Kampala, Uganda; 7 Departments of Clinical Sciences and International Public Health, Liverpool School of Tropical Medicine, Liverpool, UK; 8 WHO Global Tuberculosis Programme, World Health Organization, Geneva, Switzerland; 9 Tropical and Infectious Diseases Unit, Liverpool University Hospitals NHS Foundation Trust, Liverpool, UK

**Keywords:** Tuberculosis, Childhood, Adolescence, Socioeconomic, Life-course

## Abstract

**Background:**

Tuberculosis (TB) has been repeatedly shown to have socioeconomic impacts in both individual-level and ecological studies; however, much less is known about this effect among children and adolescents and the extent to which being affected by TB during childhood and adolescence can have life-course implications. This paper describes the results of the development of a conceptual framework and scoping review to review the evidence on the short- and long-term socioeconomic impact of tuberculosis on children and adolescents.

**Objectives:**

To increase knowledge of the socioeconomic impact of TB on children and adolescents.

**Methods:**

We developed a conceptual framework of the socioeconomic impact of TB on children and adolescents, and used scoping review methods to search for evidence supporting or disproving it. We searched four academic databases from 1 January 1990 to 6 April 2021 and conducted targeted searches of grey literature. We extracted data using a standard form and analysed data thematically.

**Results:**

Thirty-six studies (29 qualitative, five quantitative and two mixed methods studies) were included in the review. Overall, the evidence supported the conceptual framework, suggesting a severe socioeconomic impact of TB on children and adolescents through all the postulated pathways. Effects ranged from impoverishment, stigma, and family separation, to effects on nutrition and missed education opportunities. TB did not seem to exert a different socioeconomic impact when directly or indirectly affecting children/adolescents, suggesting that TB can affect this group even when they are not affected by the disease. No study provided sufficient follow-up to observe the long-term socioeconomic effect of TB in this age group.

**Conclusion:**

The evidence gathered in this review reinforces our understanding of the impact of TB on children and adolescents and highlights the importance of considering effects during the entire life course. Both ad-hoc and sustainable social protection measures and strategies are essential to mitigate the socioeconomic consequences of TB among children and adolescents.

**Supplementary Information:**

The online version contains supplementary material available at 10.1186/s12889-022-14579-7.

## Introduction

In total, an estimated 1.09 million children and young adolescents had TB in 2020 [1], with 400 000 reported, showing a significant gap in reporting [2]. TB can exacerbate poverty and social deprivation, through catastrophic health costs and reduced household income [3] and marginalised people experience a disproportionate burden of the disease. Most children develop TB as a consequence of contact with an adult family member(s) with pulmonary TB disease [4]. However, TB in the family unit may not only result in TB transmission to children, but it may also threaten household income and financial security. TB’s impacts in the household, therefore, have the potential to affect children and adolescents throughout their life course. However, there is little known about the long-term socioeconomic consequences of TB. Disruption in schooling, physical, psychological, and cognitive effects of the disease and treatment, and household poverty can impact child development, educational attainment, and economic and job prospects throughout the life course. At worst, a diagnosis of TB can spiral a family into a cycle of poverty, which can be perpetuated over generations.

Current evidence on the socioeconomic impact of TB has focused largely on adults [3], but little is known about the impact on children and adolescents, either directly when they are affected by TB, or indirectly, as household members or caregivers. There is also no theoretical understanding of these processes. Therefore, a conceptual framework was generated and scoping review was conducted to summarise and understand the evidence available on the socioeconomic impact of TB on this age group. This review sought to identify both the direct effects of TB on affected children and adolescents and the indirect effects on family units or 'households', where a primary caregiver or close family member is affected by TB. Given the limited evidence available, it was also necessary to better understand the pathways and mechanisms driving the observed socioeconomic impact of TB on children and adolescents and to ascertain which of these impacts could be targeted through structural interventions and social protection policies by national TB programmes.

### Aim

The overall aim of this review was to increase knowledge of the socioeconomic impact of tuberculosis on children and adolescents. The review had three objectives:

1. To appraise the extent to which available evidence supports a conceptual framework, defined a priori*,* on the pathways and mechanisms of socioeconomic impact beyond financial impact;
2. To better understand whether the scope of socioeconomic impact differs when the child or adolescent is the primary TB patient (i.e. TB *directly* affecting children and adolescents) as compared to another household member and/or the main caregiver (i.e. TB *indirectly* affecting children and adolescents);
3. To investigate the life-course consequences of the direct and indirect experience of TB in childhood and adolescence.

## Methods

### Conceptual framework development

**Fig. 1:**
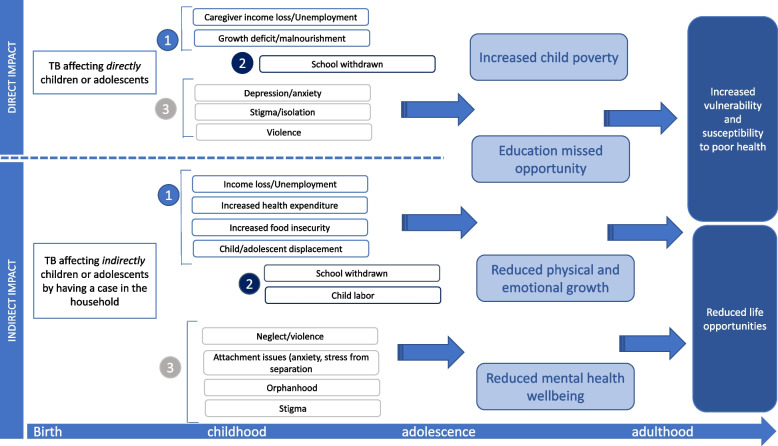
Conceptual framework of the socioeconomic impact pathways of tuberculosis (TB) on children and adolescents TB = Tuberculosis**1,2,3 refer to the material, educational and psychosocial pathways respectively, both for the direct and indirect impact. The light blue boxes are considered as outcomes and the dark blue are impact

Firstly, we developed a conceptual framework (Fig. 1) drawing on existing conceptual frameworks for tuberculosis known to the authors, and their experience in tuberculosis research (e.g. [5]) as well as theories of change for interventions for HIV prevention in children and adolescents [6, 7], complemented with expert knowledge. We searched for information on known long-term diseases affecting children (e.g. [8]) and incorporated known socioeconomic impacts from these. From these, we developed pathways through which TB may affect children and adolescents. In the conceptual framework the socioeconomic impact of TB was defined as either:

i. *direct*: when TB affects a child or adolescent in the household, or
ii. *indirect*: when TB affects other household members, and/or main caregivers.

We adopted a broad definition of socioeconomic impact encompassing the consequences of material impacts (Pathway 1 – impoverishment, e.g. [9]), educational impacts (e.g. [10]—Pathway 2 – school withdrawal), and psychosocial impacts (e.g. [11]—Pathway 3 – neglect, separation, orphanhood) (Fig. 1). All three pathways can result in child impoverishment, missed educational opportunities, impaired physical, cognitive, and emotional development, and poor mental health. If ignored, these disparities may persist and threaten onward trajectories to health and financial security in adulthood (Fig. 1). Given this, and the severity and relative chronicity of TB disease, often alongside inadequate mitigation measures, the authors adopted a life-course lens to define the socioeconomic impact of TB in childhood and adolescence. The life-course perspective [12], is defined as a multidisciplinary approach to understand the physical, mental, and social health of people, incorporating life span and life stage concepts and has not been used widely in literature on children and TB.

*Material pathway (Pathway 1)*: under both the direct and indirect routes, we anticipated that the socioeconomic impact of TB occurs via reduced income, food insecurity and loss of household income (if one of the economically active members of the household is affected by TB). These factors may, in the most extreme conditions, result in displacement of the child or adolescent to another household, withdrawal from school or child labour. When a child or adolescent is directly affected by TB this may also result in income loss for the household, as economically active household members are required to provide care. Children and adolescents may be malnourished, with the potential for stunting or wasting, due to TB itself or the secondary effects of reduced household income.

*In the educational pathway (Pathway 2),* we postulate that the effects of TB (either directly or indirectly) affect children/adolescents’ school attendance or educational development (which may in turn impact a child materially or psychosocially in the short and long-term). Poverty and malnutrition may also contribute to reduced school attendance or eventual withdrawal from school.

*In the psychosocial pathway (Pathway 3)*, we hypothesised that children and adolescents affected by TB may experience internalised stigma or discrimination, with the potential for isolation or abuse. If their main caregiver is affected by TB, there may be the potential for neglect and discrimination. Attachment may be compromised, and there is potential for separation during prolonged hospital admissions, or even through bereavement and orphanhood. These experiences are potentially traumatic and risk impaired mental health and wellbeing. The general impact and stress associated with a (relatively) chronic disease for children and adolescents with TB may also contribute to mental ill-health and affect their socioeconomic trajectory.

#### Lifecourse impacts

Through the life course lens, the impacts from all pathways may accumulate separately or interact with one another in a process of embodiment across all of these domains. For example, TB-related malnutrition may affect childhood development, causing ill health in later life. Along the education pathway, staying in education is closely tied to poverty reduction and the breaking of poverty cycles, which in turn may improve health across the life course. Not every direct consequence may occur along each pathway to every individual, but we would expect that the consequences of tuberculosis shape the inequitable distribution of socioeconomic impacts at a population level. Impacts may be short-term, medium-term, or long-term depending on the individual and the specific consequences they experience. We represent the life course as continuous in Fig. 1 from birth to adulthood to represent the ongoing nature of TB impacts across time that are not necessarily tied to the moment of illness; an individual may or may not experience reduced life opportunities immediately when diagnosed with TB, but we anticipate that these aggregate impacts accumulate to reduce life opportunities and/or increase susceptibility or vulnerability to ill health during adulthood through essentially four disadvantaged circumstances: childhood poverty, missed education opportunities, reduced physical and emotional growth and reduced mental health. These circumstances are likely to interact mutually and reinforce each other synergistically. However, the extent of their overlap and interaction is likely to differ across individuals.

### Scoping review methods

We used scoping review methodology, useful when little is published on a topic, and when addressing questions relevant to policy and practice [13]. We use the PRISMA guidelines for systematic reviews with a scoping review extension for reporting the study [14].

#### Identifying relevant studies

We searched PubMed, CINAHL, ProQuest and Scopus for studies published in peer-reviewed journals in any language from 1 January 1990 to 6 April 2021. We chose to begin our search from 1990 because the team's experience suggested the volume of research would be low before the 1990s. The search strategy was drafted in consultation with a librarian from Tampere University (See Additional file 1: Appendix 1 for a detailed description of the search strategies used). Grey literature was identified from Open Grey and Google Scholar. Additional literature was gathered through personal correspondence with key informants. Lastly, the bibliographies of the included articles were scanned, and hand searched for additional references.

#### Study selection

We included:

- primary studies
- studies that focused on children or adolescents, defined as 'children’ (9 years and below) and ‘adolescents' (10–19 years)
- studies that included any form of TB
- studies using any methods
- studies that included or focused on the social, economic, or cultural impact of TB on children or adolescents.

We excluded:

- systematic reviews or other reviews
- studies that described the development of TB vaccines or medications
- studies that described the socioeconomic causes of TB without a focus on impacts
- studies focusing only on reporting clinical outcomes or case descriptions of TB treatment

**Fig. 2:**
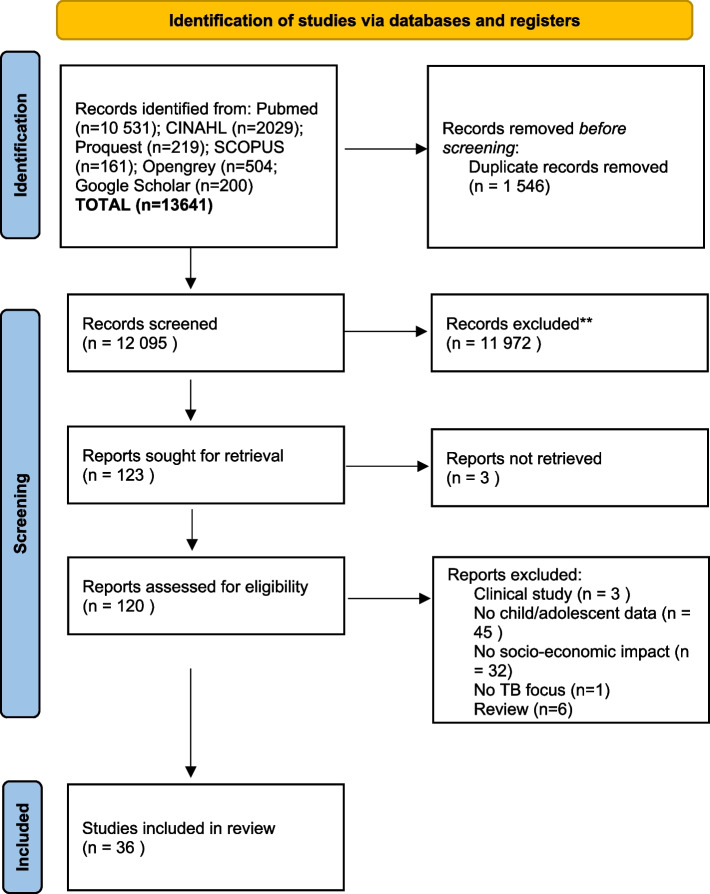
PRISMA flowchart for paper inclusion by review stage. PRISMA flowchart as described in [15]

All results were exported to Rayyan (rayyan.ai). Duplicates were removed. The studies were reviewed by the study team (SA, MRC, LH, LV, TW). Each title and abstract, where available, was reviewed by one author. Those potentially eligible (*n* = 120) were reviewed independently by two authors. The final number of studies included in the review was 36. See the PRISMA flow diagram in Fig. 2 for the details of the selection process. No quality assessment was conducted.

#### Charting the data and collating results

Data extraction on key information including aims, methods, sample and key outcomes was performed in Excel by authors independently (SA; LH; MRC; LV; DB; DC; PW; LC; TW). After extraction, the data were inductively coded by SA, LH, and MRC in Excel. We undertook a descriptive quantitative and qualitative summary to describe included studies’ characteristics with thematic analysis. Because of the life course framing of this review, we sought to draw evidence from the literature on early-life impacts as well as later-life impacts.

### Ethical considerations

Scoping reviews require no ethical review, as they include secondary, published data. However, given that scoping reviews may be used to influence policy or the identification of research gaps, they require transparency in both analysis and data collection to ensure that included studies have robust ethical and methodological foundations, and biases are identified and acknowledged. In this study, we also sought to use non-stigmatizing language [16].

## Results

### Descriptive analysis of included studies

See Table 1 for study characteristics. We included 36 papers. In the African region (n = 17) studies were from South Africa (*n* = 10), Ghana (*n* = 2) and one each from Botswana, Lesotho, Nigeria, Uganda, and Zambia. South-East Asia was represented by eight studies, specifically from India (*n* = 4), Nepal (*n* = 2), Bangladesh (*n* = 1) and Thailand (*n* = 1). Five studies were from the Americas, Peru (*n* = 3), Brazil (*n* = 1), and Venezuela (*n* = 1). Five papers were from the Western Pacific region, specifically China (*n* = 3), Malaysia (*n* = 1) and Vietnam (*n* = 1). There was one Egyptian study from the Eastern Mediterranean region. Thirty-five studies were published in English and one [17] in Spanish.

**Table 1:** Characteristics of included studies

Article short name	Country	WHO region	Method	Type of TB
Atsa Dodor & Kelly 2009 [18]	Ghana	Africa	Qualitative	Not specified
Awaluddin et al. 2020 [19]	Malaysia	Western Pacific	Qualitative	Not specified
Baral et al. 2007 [20]	Nepal	South-east Asia	Qualitative	Not specified
Barua et al. 2018 [21]	Bangladesh	South-east Asia	Qualitative	Not specified
Buregyeya et al. 2011 [22]	Uganda	Africa	Qualitative	Not specified
Coit et al. 2020 [23]	Peru	Americas	Qualitative	Pulmonary TB
Cremers et al. 2015 [24]	Zambia	Africa	Mixed methods	EPTB or PTB
Dodor 2012 [25]	Ghana	Africa	Qualitative	Not specified
van Elsland et al. 2012 [26]	South Africa	Africa	Qualitative	TB meningitis
Franck et al. 2014 [27]	South Africa	Africa	Qualitative	RR TB
Ganapathy et al. 2008 [28]	India	South-East Asia	Qualitative	Not specified
Goudge et al. 2009 [29]	South Africa	Africa	Mixed methods	Any TB
Goyal-Honavar et al. 2020 [30]	India	South-east Asia	Qualitative	Not specified
Hirsch-Moverman et al. 2020 [31]	Lesotho	Africa	Qualitative	Not specified
Hutchinson et al. 2017 [32]	China	Western Pacific	Qualitative	RR TB
Krauss-Mars & Lachman 1992 [33]	South Africa	Africa	Quantitative	TB meningitis
Lewis & Newell 2009 [34]	Nepal	South-east Asia	Qualitative	Not specified
Lohiniva et al. 2016 [35]	Egypt	Eastern Mediterranean	Qualitative	Any TB
Long et al. 2001 [36]	Vietnam	Western Pacific	Qualitative	Smear-positive pulmonary TB
Loveday et al. 2018 [37]	South Africa	Africa	Qualitative	RR TB
de Carvalho Machado et al. 2015 [38]	Brazil	Americas	Qualitative	Any TB
Masuku et al. 2018 [39]	South Africa	Africa	Qualitative	Not specified
Maurera et al. 2019 [17]	Venezuela	Americas	Qualitative	Any TB
McNally et al. 2019 [40]	Peru	Americas	Qualitative	RR TB
Ngamvithayapong-Yanai et al. 2005 [41]	Thailand	South-east Asia	Qualitative	New TB diagnoses
Onazi et al. 2015 [42]	Nigeria	Africa	Quantitative	Any TB
Paz-Soldan et al. 2013 [43]	Peru	Americas	Qualitative	Not specified
Rajeswari et al. 1999 [44]	India	South-east Asia	Quantitative	PTB
Schoeman et al. 2002 [45]	South Africa	Africa	Quantitative	TB meningitis
Skinner et al. 2013 [46]	South Africa	Africa	Qualitative	TPT
Stillson et al. 2016 [47]	Botswana	Africa	Qualitative	Not specified
Wait & Schoeman 2010 [48]	South Africa	Africa	Quantitative	TB meningitis
Westaway & Wessie 1994 [49]	South Africa	Africa	Qualitative	Not specified
Yellappa et al. 2016 [50]	India	South-east Asia	Qualitative	Not specified
Zhang et al. 2014 [51]	China	Western Pacific	Qualitative	Not specified
Zhang et al. 2016 [52]	China	Western Pacific	Qualitative	Not specified

*EPTB* Extra-pulmonary TB, *TPT* TB preventive treatment, *LMIC* Low- and middle-income countries, *RR-TB* Drug-resistant tuberculosis, *PTB* Pulmonary TB, *TB* Tuberculosis

Studies utilized mainly qualitative methods (*n* = 29). Five were quantitative and two were mixed methods studies. Most (*n* = 25) studies did not specify the type of TB that study participants had or described it in general terms. Studies that described participant disease characteristics, included TB meningitis (*n* = 4), pulmonary TB (*n* = 3) and drug-resistant TB (RR-TB; *n* = 4). Half of the studies included children as their main focus, and half focused on TB in the family or community but included findings on the socioeconomic impact on children or adolescents.

Thirteen studies did not specify the age of children or adolescents included. Three studies focused on children aged nine years and below, five on adolescents between 10 to 19 years of age; and fourteen on both adolescents and children. The studies reported varied sample sizes (from 3–1446 individuals). The studies reported on 520 interviews and 42 focus group discussions.

### Summary of overall quantitative findings

Five studies provided quantitative data (Table 2). Except for one [44], all were based in sub-Saharan Africa. All studies adopted a cross-sectional approach, with evidence from *ad-hoc* surveys in the study population. In three studies, mixed methodology was used [24, 42, 44]. In total, 50% of the papers reported evidence of TB directly involving children and adolescents [24, 45, 48]; however, the sample size and age of children/adolescents were not necessarily specified. It was therefore difficult to fully establish the different effects experienced by children as compared to adolescents, and difficult to make inferences about the robustness of these study findings based on sample size.

**Table 2:** Quantitative studies description

Author/year	Country	Study methods	Study objective	TB impact (direct or indirect	Children or adolescents	Number of children/adolescents reported	Age of children (range)
Onazi / 2015 [42]	Nigeria	Mixed methods	Addressing economic and societal consequences of TB on patients and their household members	Indirect	NA	NA	NA
Rajeswari / 1999 [44]	India	Mixed methods	Assessing socioeconomic constraints faced by TB patients	Indirect	Children and adolescents	276 (only for the assessment on education)	6–16
Cremers / 2015 [24]	Zambia	Mixed methods	To enhance understanding of TB-related stigmatizing perceptions and to describe TB patients’ experiences of stigma	Direct	Children and adolescents	25	1–19
Schoeman / 2002 [45]	South Africa	Quantitative	To determine the long-term outcome of children diagnosed with TB meningitis and treated with modern antituberculosis drugs	Direct	Children	76	Median age at admission 29 months and 9 years at follow up
Wait / 2010 [48]	South Africa	Quantitative	To investigate child behaviour in children who recovered from tuberculous meningitis (TBM)	Direct	Children	74	10 years and 7 months median age at evaluation

All types of socioeconomic impact (i.e. financial, educational, and psychosocial) were noted in both the direct and indirect TB impact domain (Table 3). The socioeconomic impact of TB was negative across all studies. While a comparison group was often lacking, there were examples of direct quantitative impacts from Schoeman et al. (2002): 80% of children experienced cognitive impairment, 43% experienced poor scholastic progress, and 40% experienced emotional disturbance, reported in a study on TB meningitis [45]. Of concern, longer-term cognitive and behavioural sequelae were reported for children affected by TB meningitis [45, 48]. Two studies reported a direct impact of school withdrawal in 2.6 and 11% of children and adolescents, respectively [42, 44]. In one Indian study [44], 8% of children had to start working to support the family during the TB episode in the household as described in the indirect educational pathway.

**Table 3:** Quantitative socioeconomic impact (A) and (B)

(A) Socioeconomic impact on children/adolescents *directly* affected by TB					
Author	Impact (1)	Impact (2)	Impact (3)	Pathway on the Conceptual framework (4)	Recommendations by the authors (5)
Cremers / 2015 [24]	Psychosocial—stigma	Negative	81.9% of TB patients encountered consequences of stigma Quantitative data demonstrated that children were as vulnerable as adults to suffer from the social consequences of stigma (Not presented in the paper)	Direct/3	Implementation of interventions at reducing stigma
Schoeman / 2002 [45]	Education	Negative	Cognitive impairment (80%), poor scholastic progress (43%), and emotional disturbance (40%). Twenty-two per cent of parents complained that their children were aggressive, restless, hyperactive, or had a low level of tolerance to frustration	Direct/1 and Direct/2	It was concluded that these disabilities in children from mainly deprived socioeconomic backgrounds have serious implications for their future social, academic, and career prospects
Wait /2010 [48]	Education / psychosocial mental health	Negative	TBM survivors overall showed elevated scores related to social problems, somatic complaints, and total problems. More severe cases had the worst scores on scales that measure disruptive behaviour, attention problems, hyperactivity, aggressive behaviour, rule-breaking behaviour, social behaviour problems, conduct problems and somatic complaints. These findings may suggest a high incidence of ADHD or conduct disorder amongst more severe cases of TB meningitis, however, also this group had a significantly lower mean IQ when compared to less severe cases	Direct/2 and Direct/3	Authors conclude that intellectual deficit, general behavioural disinhibition as well as internalized emotional disorder are common long-term complications of the survivors of TBM, especially among severe (Stage III) patients
(B) Socioeconomic impact on children/adolescents *indirectly* affected by TB					
Author	Impact (1)	Impact (2)	Impact (3)	Map on the Conceptual framework (4)	Recommendations by the authors (5)
Onazi/2015 [42]	Education	Negative	2.6% of respondents dropped out of school or had a sick child because of TB	Indirect TB/ 2	Loss of income by TB patients should be addressed through better labour protection policies
Rajeswari/1999 [44]	Education, psychosocial, financial	Negative	• Thirty-four per cent of patients reported that due to loss of income they could not afford to buy adequate food or clothing or books for their children • Female patients reported their inability to care for their children and to perform routine household activities • Eleven per cent of the children had discontinued school because of the burden caused by the parent’s illness (8% rural vs. 13% urban, *P* = 0.05) • 23 children (8%) took up employment to support the family	Indirect TB/1,2,3	Better support for TB patients, especially women

(1) Type of impact (financial, psychosocial etc.)

(2) Impact observed (positive, negative, null)

(3) Impact observed description (if quantitative percentages, odds ratio etc., if qualitative the actual response provided)

(4) Pathway in the conceptual framework that is supported by this evidence

We attempted to examine whether impacts differed across studies’ design or study population (i.e. direct or indirect), but due to the limited number of quantitative studies, we could not detect any relevant difference (i.e. in terms of magnitude and/or direction of effect).

Even if often in generic terms, most studies concluded by providing recommendations, summarized as the need for better financial and psychosocial support for TB-affected households; further, there is consensus that socioeconomic effects observed are likely to produce long-term consequences on children and adolescents and thus are to be averted or at least mitigated.

### Analysis of qualitative and mixed methods studies

The qualitative findings suggest that experiencing TB during childhood or adolescence (whether as a patient or as a household member of a person with TB) appears to have mainly negative impacts.

#### Financial impact of TB: spending, nutrition, and education (Pathway 1)

Twelve papers described the financial impact of childhood or adolescent TB or TB affecting a household member. The financial consequences of TB were described as impacting multiple aspects of family life, causing anxiety, and impacting directly on child and adolescent nutrition and education [35, 37, 46, 52]. While the financial impact resulted from direct and indirect costs of seeking and attending treatment, TB also caused a loss of income for the entire family due to caring responsibilities and treatment requirements, such as travel to the hospital [26].

Being affected by TB oneself, or administering TB treatment for children, at home or in a clinical setting, restricted the possibility of those affected or their caregivers to earn a wage [23, 37, 43, 47, 51], which impacted finances. Those personally affected by TB often had to rely on others for income when they were not able to work, as described in a study conducted among migrants in Egypt [35]. The combination of being affected by TB oneself, while trying to manage a child’s TB diagnosis, and earn an income was noted to be especially difficult in Peru [23]. The costs of travel to the hospital, food and access to healthcare (e.g. travel to hospital or clinic) were frequently mentioned as causes of financial distress [27, 29, 37, 47]. These costs were particularly difficult to manage when adults were unemployed [37].

Reduced household income and catastrophic health costs, and their impact on children caused anxiety among parents in South Africa [37] and China [51] and resulted in negative coping and borrowing of money. In China, adolescents also reported anxiety over their family’s financial situation due to treatment [52]. While noting that families bore the most responsibility for financial support, patients suggested that additional economic support should be made accessible in Peru [43].

#### TB impacting children's education (Pathway 2)

Seven papers described the impact of TB on children’s education. Papers indicated generally that the impact of TB on the family involved all spheres of life, including income, health, education and nutrition [30, 44]. The impact on children’s education was perceived to be stronger when the person with TB was a male wage earner in India [44].

The impact of TB on children’s education related to the disease itself and TB treatment requirements on academic performance and behaviour [27, 37]. In South Africa, parents described behavioural, emotional and cognitive difficulties after RR-TB diagnosis, that also impacted academic performance [37].

Hospitalisation [37] and the need to collect medication during school hours [50] also disrupted education. In South Africa, at least one child had been dismissed from school due to the stigma of TB [37], while in China adolescents could not return to school during the first two months of TB treatment [52], despite this being against doctors’ recommendations. However, children in China [52] also reported enjoying attending hospital school. One Brazilian study described a child being withdrawn from school because a parent had “stopped everything” that had not been related to treating TB [38].

Disrupted education was reported to be stressful, especially by two papers from China that focused on adolescent and parental experiences of TB [51, 52]. In this instance, affected adolescents were enrolled in an academic year with exams that would impact their future higher education. Parents and children were anxious that the lapse in school attendance due to unplanned illness would impact their college scores and exam results, with long-term significance for their career prospects and prosperity.

However, in circumstances where education was interrupted, a study suggested that children re-integrated relatively quickly despite some initial challenges [37].

#### Stigma: not a uniform experience, but experienced by many (Pathway 3)

Fifteen qualitative papers discussed stigma and were from Peru, Brazil, Botswana, South Africa, Lesotho, Ghana, Vietnam, Nepal, China, and India.

Stigma was noted in China for people with RR-TB [32], and people with TB in Nepal [20] and Peru [43]. Not all adolescents experienced stigma [39] even if they disclosed their treatment condition [20]. Examples of enacted stigma, and discrimination due to a real or perceived TB diagnosis, included a report from Peru [43] where patients, including children, were excluded from church. Children also experienced discrimination from parents preventing their children from playing with children from families with TB in Peru [43], South Africa [37] and Uganda [22]. Anticipated stigma, individuals believing others will discriminate against them, caused worry and anxiety among parents, in studies from South Africa [37, 49].

Stigma had practical implications for diagnosis, clinic attendance and treatment. In Botswana [47], respondents indicated that those with TB should not collect their medication from the same place as the general public. Skinner et al. [46] in South Africa noted that healthcare workers believed that the combined stigma of HIV and TB may have meant that parents refused TB preventive treatment for their children. In Lesotho, it was noted also that anticipated stigma may prevent parents from bringing their children for preventive treatment [31]. Supporting this, in Brazil, it was specifically noted that stigma could contribute to children’s non-adherence to treatment [38].

Stigma also seemed to be gendered. Several papers reported worse TB-related stigma for women than for men, which could also apply to younger age groups, particularly at the age of marriage. Reports from Vietnam [36], India [28] and Ghana [25] suggested that TB and stigma could impact a girl's marital prospects. Long et al. [36] suggested this was due to social stigma; while in India [28] and Ghana [25], community perceptions indicated fear of infection of the partner or children from the marriage.

#### Psychosocial impacts: TB affects parenting and childcare, and causes separation (Pathway 3)

Several papers (*n* = 13) discussed the family and community support required by families with TB, and TB’s social impact on families.

Two articles reported positive impacts of TB mitigation strategies on patients’ social wellbeing. Zhang in China [52] reported a positive impact of social support for adolescents, with friends calling them to see how they were when not at school. In Peru, [43] patients had expanded their social network due to TB support groups, which could impact children indirectly. In Malaysia, community members thought that community support could help TB patients to adhere to treatment [19].

Within the household, TB also affected family relationships. TB in the family had resulted in the breakdown of parental relationships [18, 37] in two studies, likely to affect the well-being of children. The studies indicated one possible cause for this was increased household stress, and indeed, parental stress due to the social and economic implications of TB [40, 49, 51]. Studies also mentioned further strain on family relationships from parental guilt from the possibility of TB transmission to their child [40, 49, 51] or others [37].

Fear of transmission resulted in the separation of children from their parents [18, 21, 35, 36, 40, 41]. One South African study reported parental death from TB and abandonment of children due to TB [37], while another described a child abandoned by a mother in the context of TB, in Venezuela [17]. Although separation appeared not to be mandated by health services, parents and families often made voluntary arrangements to ensure that children were not in close contact, or sharing utensils, with their affected parent. Separation was also caused by hospitalization.

Partly due to separation, or due to parental illness from tuberculosis, TB in the family influenced how and by whom children were cared for in Ghana, China, and Nepal [25, 32, 34, 52]. In China, several adolescents reported a sense of resentment due to increased reliance and dependency on their parents during their illness [52].

### Gaps in evidence: comparing the conceptual framework with located evidence

Overall, most pathways seem to be supported by the evidence identified. However, the data included in this review were too limited to distinguish between the multiple possible theoretical pathways. An absence of pathway-specific effect estimates in the quantitative data and sufficiently rich description in the qualitative data means we cannot fully support every pathway in the conceptual framework. The pathways in the conceptual framework should be investigated further.

There seems to be no major difference between the pathways involved in both the direct and indirect impact effect of TB: most pathways are documented in both domains. The evidence was also not balanced in terms of the volume of evidence on each topic – stigma is an area that has been researched extensively, and we could find far more evidence for its effects, than that of, for example, education. There were also disparities in how the financial impact of TB was represented. The financial impact of TB can result in catastrophic costs for households (e.g. [3, 53]), however, information in the studies included was not specific to children and adolescents, suggesting a need for disaggregation and a better understanding of TB's financial impact on children and adolescents. There were no new impacts that we discovered in our comprehensive search of the literature, suggesting that the conceptual framework captured impacts adequately. However, research is needed to assess the reciprocal importance of each pathway, and their interaction with each other, to identify the most appropriate ‘entry points’ for interventions.

## Discussion

To our knowledge, this is the first attempt to systematically appraise the socioeconomic impact of TB specifically on children and adolescents. We deliberately adopted a broad socioeconomic approach, encompassing domains other than financial ones. This was dictated by the need to encompass factors that were likely to impact the socioeconomic life trajectories of children (i.e. by making them more vulnerable (either physically or emotionally) or by reducing their access to life opportunities (in terms of education and future employability). We deemed this life-course perspective essential to capture fully the socioeconomic impact of TB in this distinct age group. This review indicates that TB impacts negatively on the economic and psychosocial well-being of children, adolescents, and their families. TB could affect children directly, for example by limiting educational possibilities, or indirectly, by causing family distress through reduced finances, separation from parents, or discrimination of entire families due to TB. The nature of the studies makes it difficult to separate the direct impact on children from the impacts on family, but given the general closeness of family units, it is plausible that family distress impacts also children. For example, the studies included indications of practices such as separating children from parents, that could be harmful at key developmental stages. However, as the studies were cross-sectional, and included little detail on the children, we cannot know the long-term effects of these practices.

Financial distress was clear from the studies and had a clear direct impact on children’s nutrition and education. It was evident that parents, frequently mothers, had to give up work to care for their children with TB [33]. Financial effects were also more severe for those who were unemployed [37]. Maintaining household income was made more difficult by clinic opening times that often conflicted with working hours [47]. The challenges in prioritizing TB treatment and care for families and individuals are well established [54]. One study emphasised the role of ‘home care’, which was more flexible, and allowed parents to combine caring for their children with employment [26]. In combination with the hidden costs of visiting children in hospital, home care with adequate medical and social support may be a potential strategy to reduce the financial strain on households and reduce parental stress and anxiety. This could also be in line with the patient-centred treatment focus of the End TB Strategy [55]. However, transfers of care from health centres to the home should only be done with adequate support for the parents or caregivers.

The evidence retrieved suggested that TB impacts on children's or adolescents' education, either through children being excluded from school or being too unwell to attend; or having to take up work or give up school due to financial struggles. TB can impact children and adolescents at critical periods, during preparation for ‘final’ or ‘exit’ exams that may contribute to their perceived educational attainment and onward career choice [51, 52]. Examples of altered behaviour and/or cognition following TB meningitis [37] are of equal concern, potentially impacting children's lives in the long term. Policies to support children and adolescents should include supporting them to maintain schooling while they are being treated for TB. This is a challenge when TB is not well understood by school leaders, as some children are not allowed to return to school while on TB treatment [37, 52], even when they are no longer infectious [52].

Stigma among people with TB has been extensively studied and is thought to contribute to diagnostic delay and treatment non-adherence [56]. Stigma was identified in our review for those requiring TB preventive treatment in childhood, in an HIV endemic area [46], and limiting children’s social interactions with other children. Addressing stigma at a community level is needed, including increased education among communities about how TB may be spread. Stigma may also be internalized by people with TB, which can contribute to anxiety. Initial reports suggest that TB clubs and peer support can be useful for reducing internalized stigma [57].

The findings of our review also emphasise the interconnected nature of family units, even when one person is affected by TB in the household. If the person affected earns the primary household income, the negative impact is worse for children [44]. Further analysis of these issues could be gained from tuberculosis patient cost surveys [53]. Beyond cost, if the person affected by TB is the mother, this impacts household dynamics and caregiving arrangements [58]. In addition, evidence suggests that TB may contribute to the breakdown of parental relationships, and consequently the family unit. These factors may all have profound effects on children or adolescents. The loss of a parent may predispose a child to poverty and lower educational attainment [59]. However, promisingly we found no evidence of child abuse and neglect in the studies included, or any evidence of alcohol or other substance use within affected households.

This review’s key strengths include the life-course perspective, inclusion of several databases, and the international multidisciplinary team involved in the study. However, from a conceptual perspective there are two key limitations to our findings: 1. no study provides sufficient evidence to support the long-term/life-course perspective we postulated in this review. While this lens is often assumed and authors mention the long-term consequences of TB, studies are not designed to capture this effect properly and thus, while highly plausible, the life-course perspective cannot currently be demonstrated 2. Despite the negative impact of TB on children and adolescents, few studies provide recommendations or possible solutions to compensate for or reverse this. Further limitations include a lack of disaggregated data and a limited focus on children and adolescents overall.

Given our analysis of the included studies, we have generated a set of suggestions for the way forward (see Table 4 below).

**Table 4:** Suggested actions in further research, policy and practice

This review suggests that:
1. More studies are required to evaluate the broader socioeconomic impacts of TB on children, adolescents and family units across different settings, with the comparison of effects according to different age groups both in the short- and the long-term;
2. There is a need for standardisation of age groups as well as outcomes (including income measurement) to allow for comparison of outcomes for different age categories globally, alongside the disaggregation of all studies in terms of affected age groups to allow for responsive mitigation strategies, for example analysing groups 0 < 5, 5 < 10, 10 < 14 and 14 < 19 separately;
3. Onward studies could adopt prospective designs to best capture the long-term socioeconomic consequences of TB, during childhood and adolescence, and to better ascertain whether effects are permanent or reversible over time. This may allow social protection strategies to be implemented to mitigate the potential life-course impact of TB on children and adolescents
4. Future studies will have to be designed to clearly distinguish between the direct and indirect effects of TB on children and adolescents. Nonetheless, our review suggests that TB affects the whole family as a unit with different consequences among all its members. This complexity should be considered when designing strategies and policies to mitigate the short- and long-term consequences of TB
5. More formative research and consideration of treatment strategies that minimize the risks of exacerbating poverty during anti-TB treatment and post-TB-impacts, while carefully weighing their potential burden to households, with household support strategies that may include social protection; and
6. Further research should be conducted on how different social protection programmes (in-kind, cash transfer, or cash + approaches) impact the short- or long-term consequences of TB as well as how existing programmes could become more sensitive toward people with TB and how child-sensitive social protection could better support families and people with TB better

## Conclusion

We identified 36 studies, globally, evaluating the socioeconomic impact of TB among children and adolescents, and found that TB impacts the well-being of children, adolescents, and families. The life course impact of TB on children and adolescents is plausible: the type of impact that was reported (either financial, psychosocial and educational) either directly or indirectly can potentially change the life course trajectory of these individuals. None of the included studies, however, could fully demonstrate this, because of the lack of longitudinal design and follow-up data. We found the socioeconomic impacts to be mainly negative, relating to the financial, psychosocial, and educational wellbeing of children, adolescents, and families. More high-quality, longitudinal research is needed on the long-term impact of these effects on the life-course of children and adolescents, to fully understand the life-course consequences of TB.

## Supplementary Information

**Additional file 1:** **Appendix 1.** Search strategy. 

## Data Availability

The data are available from the corresponding author (*salla.atkins@tuni.fi*) upon reasonable request.

## Abbreviation

TB: Tuberculosis
