# Supplementary material for: The socioeconomic impact of tuberculosis on children and adolescents: a scoping review and conceptual framework
Source: BMC Public Health. 2022 Nov 23;22:2153. doi: 10.1186/s12889-022-14579-7 (PMC9686126; doi:10.1186/s12889-022-14579-7)
Supplement: Supplementary file 1 — Additional file 1: Appendix 1. Search strategy. [file 12889_2022_14579_MOESM1_ESM.docx]

# Appendix 1- Search strategy

## PubMed

| **Search #** | **Search terms** |
| --- | --- |
| **#1 Tuberculosis** | TI(Tubercul* OR TB OR antitubercular agents OR ltbi) |
| **#2** | AB(Tubercul* OR TB OR antitubercular agents OR ltbi) |
| **#3 Child/adolescent** | TI(child* OR adolescen* OR famil* OR mother* OR father* OR young people OR youth OR teenage* OR caregiver* OR parent* OR minors) |
| **#4** | AB(child* OR adolescen* OR famil* OR mother* OR father* OR young people OR youth OR teenage* OR caregiver* OR parent* OR minors) |
| **#5 Socioeconomic impacts** | TI(socioecono* OR socio-econo* OR poverty OR orphan* OR malnourish* OR food insecur* OR stigma* OR displace* OR attach* OR stress* OR anxiety OR unemploy* OR undernourish* OR school* OR isolation* OR violen* OR neglect OR income loss OR growth* OR education* OR work* OR labour* OR labor OR death OR developmental delay OR discrimination OR socioeconomic impact* OR socio-economic impact* OR Quality of Life OR Health Expenditures OR Cost of Illness OR Child Abuse OR “Child to Parent Abuse” OR Parent-Infant-Child Attachment OR Child Mortality OR Child Labor OR Infant Death) |
| **#6** | AB(socioecono* OR socio-econo* OR poverty OR orphan* OR malnourish* OR food insecur* OR stigma* OR displace* OR attach* OR stress* OR anxiety OR unemploy* OR undernourish* OR school* OR isolation* OR violen* OR neglect OR income loss OR growth* OR education* OR work* OR labour* OR labor OR death OR developmental delay OR discrimination OR socioeconomic impact* OR socio-economic impact* OR Quality of Life OR Health Expenditures OR Cost of Illness OR Child Abuse OR “Child to Parent Abuse” OR Parent-Infant-Child Attachment OR Child Mortality OR Child Labor OR Infant Death) |
| **#7** | #1 AND #3 AND #5 |
| **#8** | #2 AND #4 AND #6 |
| **#9** | #7 OR #8 |
| **Filters** | Year published: Between January 1^st^, 1990 through April 1^st^, 2021 |

**Proquest**

**Medline (PubMed)**

| **Search #** | **Search terms** |
| --- | --- |
| **#1 Tuberculosis** | (Tubercul*[Title/Abstract] OR TB[Title/Abstract] OR ltbi[Title/Abstract]) OR (Tuberculosis OR Tuberculosis, Multidrug-Resistant OR Antitubercular Agents[MeSH Terms]) |
| **#2 Socioeconomic impacts** | (socioecono*[Title/Abstract] OR socio-econo*[Title/Abstract] OR poverty[Title/Abstract] OR orphan*[Title/Abstract] OR malnourish*[Title/Abstract] OR food insecur*[Title/Abstract] OR stigma*[Title/Abstract] OR displace*[Title/Abstract] OR attach*[Title/Abstract] OR stress*[Title/Abstract] OR anxiety[Title/Abstract] OR unemploy*[Title/Abstract] OR undernourish*[Title/Abstract] OR school*[Title/Abstract] OR isolation*[Title/Abstract] OR violen*[Title/Abstract] OR neglect[Title/Abstract] OR income loss[Title/Abstract] OR growth*[Title/Abstract] OR education*[Title/Abstract] OR work*[Title/Abstract] OR labour*[Title/Abstract] OR labor[Title/Abstract] OR death[Title/Abstract] OR discrimination[Title/Abstract] OR development delay[Title/Abstract] OR Child Abuse[Title/Abstract] OR "Child to Parent Abuse"[Title/Abstract] OR "Parent-Infant-Child Attachment"[Title/Abstract] OR Child Mortality[Title/Abstract] OR Child Labor[Title/Abstract] OR Infant Death[Title/Abstract]) OR ("Socioeconomic Factors" OR "Adverse Childhood Experiences" OR "Quality of Life" OR "Health Expenditures" OR "Cost of Illness" OR "Child Abuse" OR "Family Separation" OR "Juvenile Delinquency" OR "Death"[MeSH Terms]) |
| **#3 Child/adolescent** | (famil*[Title/Abstract] OR mother*[Title/Abstract] OR father*[Title/Abstract] OR young people[Title/Abstract] OR youth[Title/Abstract] OR teenage*[Title/Abstract] OR caregiver*[Title/Abstract] OR parent*[Title/Abstract] OR minors[Title/Abstract]) OR ("Child" OR "Adolescent" OR "Infant" OR "Family" OR "Parents" OR "Family Health" OR "Caregivers"[MeSH Terms]) |
| **#4** | #1 AND #2 AND #3 |
| **Filters** | Year published: Between January 1^st^, 1990 through April 6^th^, 2021 |

**Scopus**

| **Search #** | **Search terms** |
| --- | --- |
| **#1 Tuberculosis** | tuberculosis OR tb OR ltbi OR "antitubercular agents" |
| **#2 Child/adolescent** | child OR adolescent OR adolescence OR family OR mother OR father OR “young people” OR youth OR teenager OR caregiver OR parent OR parental OR minors |
| **#3 Socioeconomic impacts** | Socioeconomic OR socio-economic OR poverty OR orphan OR malnourish OR “food insecure” OR “food insecurity” OR stigma OR displacement OR attachment OR stress OR anxiety OR unemployment OR displacement OR attachment OR stress OR anxiety OR unemployment OR undernourished OR school OR isolation OR violent OR violence OR neglect OR “income loss” OR growth OR education OR work OR labour OR labor OR death OR “developmental delay” OR discrimination OR “socioeconomic impact” OR “socio-economic impact” OR “Quality of Life” OR “Health Expenditures” OR “Cost of Illness” OR “Child Abuse” OR “Child to Parent Abuse” OR “Parent-Infant-Child Attachment” OR “Child Mortality” OR “Child Labor” OR “Infant Death” |
| **#4** | #1 AND #2 AND #3 |
| **Filters** | TITLE-ABS-KEY  Year published: Between January 1^st^, 1990 through April 7^th^, 2021 |

**CIHNAL**

| **Search #** | **Search terms** |
| --- | --- |
| **#1 Tuberculosis** | TI (tuberculosis or tb or latent tuberculosis infection or ltbi OR (MH "Tuberculosis, Multidrug-Resistant") OR antitubercular agents OR (MH "Tuberculosis, Osteoarticular+") OR (MH "Tuberculosis, Pulmonary") OR (MH "Mycobacterium Tuberculosis") OR (MH "Tuberculosis, Meningeal")) |
| **#2** | AB (tuberculosis or tb or latent tuberculosis infection or ltbi OR (MH "Tuberculosis, Multidrug-Resistant") OR antitubercular agents OR (MH "Tuberculosis, Osteoarticular+") OR (MH "Tuberculosis, Pulmonary") OR (MH "Mycobacterium Tuberculosis") OR (MH "Tuberculosis, Meningeal")) |
| **#3 Socioeconomic impacts** | TI (socioecono* OR socio-econo* OR poverty OR orphan* OR malnourish* OR food insecur* OR stigma* OR displace* OR attach* OR stress* OR anxiety OR unemploy* OR undernourish* OR school* OR isolation* OR violen* OR neglect OR income loss OR growth* OR education* OR work* OR labour* OR labor OR death OR discrimination OR development delay OR (MH "Child Abuse") OR (MH "Child to Parent Abuse") OR (MH "Risk for Altered Parent-Infant-Child Attachment (NANDA)") OR (MH "Child Mortality") OR (MH "Child Abuse, Sexual") OR (MH "Child Labor") OR (MH "Infant Death") OR (MH "Quality of Life") OR "health expenditures" OR (MH "Economic Aspects of Illness") ) |
| **#4** | AB (socioecono* OR socio-econo* OR poverty OR orphan* OR malnourish* OR food insecur* OR stigma* OR displace* OR attach* OR stress* OR anxiety OR unemploy* OR undernourish* OR school* OR isolation* OR violen* OR neglect OR income loss OR growth* OR education* OR work* OR labour* OR labor OR death OR discrimination OR development delay OR (MH "Child Abuse") OR (MH "Child to Parent Abuse") OR (MH "Risk for Altered Parent-Infant-Child Attachment (NANDA)") OR (MH "Child Mortality") OR (MH "Child Abuse, Sexual") OR (MH "Child Labor") OR (MH "Infant Death") OR (MH "Quality of Life") OR "health expenditures" OR (MH "Economic Aspects of Illness")) |
| **#5 Child/adolescent** | TI (child* OR adolescen* OR famil* OR mother* OR father* OR young people OR youth OR teenage* OR caregiver* OR parent* OR (MH "Single Parent") OR (MH "Caregivers") OR (MH "Caregivers") OR (MH "Parents") OR (MH "Child") OR (MH "Adolescence") OR (MH "Minors (Legal)") |
| **#6** | AB (child* OR adolescen* OR famil* OR mother* OR father* OR young people OR youth OR teenage* OR caregiver* OR parent* OR (MH "Single Parent") OR (MH "Caregivers") OR (MH "Caregivers") OR (MH "Parents") OR (MH "Child") OR (MH "Adolescence") OR (MH "Minors (Legal)") |
| **#7** | 1# AND #3 AND #5 |
| **#8** | 2# AND #4 AND #6 |
| **#9** | #7 OR #8 |
| **Filters** | Year published: Between January 1^st^, 1990 through April 7^th^, 2021 |

**Open grey**

| **Search #** | **Search terms** |
| --- | --- |
| **#1 Tuberculosis** | tuberculosis |
| **#2** | tuberculosis discipline:(06E - Medicine) |
| **#3** | tuberculosis discipline:(060 - Biological and medical sciences, general) |
| **#4** | tuberculosis discipline:(05T - Health services, health administration, community care services) |

**Google Scholar**

| **Search #** | **Search terms** |
| --- | --- |
| **#1** | +tuberculosis +child +(impact or effect) +(social OR economic) -vaccine -genome -vaccination |
| Filters | From 1990 to 2021  Limit to 20 first pages |
